# Improved gonadal hormones levels following 6 and 12 months of anti-retroviral therapy among men living with human immunodeficiency virus infection

**DOI:** 10.1371/journal.pone.0324374

**Published:** 2025-05-28

**Authors:** Shabani Iddi, Haruna Dika, Karol J. Marwa, Benson R. Kidenya, Samuel E. Kalluvya

**Affiliations:** 1 Department of Physiology, Weill Bugando School of Medicine, Catholic University of Health and Allied Sciences, Mwanza, Tanzania; 2 Department of Pharmacology, Weill Bugando School of Medicine, Catholic University of Health and Allied Sciences, Mwanza, Tanzania; 3 Department of Biochemistry and molecular biology, Weill Bugando School of Medicine, Catholic University of Health and Allied Sciences, Mwanza, Tanzania; 4 Department of Internal Medicine, Weill Bugando School of Medicine, Catholic University of Health and Allied Sciences, Mwanza, Tanzania; General Sir John Kotelawala Defence University Faculty of Medicine, SRI LANKA

## Abstract

**Background:**

Male hypogonadism is commonly reported in men living with human immunodeficiency virus (HIV) (MLWH), reaching a prevalence of up to 89% and shown to decrease in the antiretroviral therapy (ART) era as compared to pre-ART era. Data regarding the effect of ART on gonadal hormones levels are scarce. We aimed to determine changes in gonadal hormones levels in HIV males following ART initiation.

**Methods:**

This was a longitudinal study involving newly diagnosed ART naïve MLWH in Mwanza, Tanzania. All enrolled participants underwent thorough clinical and physical examination including anthropometric measurements. A pre-structured questionnaire was used to collect socio-demographic and clinical data. Serum total testosterone (TT), follicle-stimulating hormone (FSH), luteinizing hormone (LH) and estradiol were estimated at baseline, six and twelve months after ART initiation. Serum TT < 300 ng/dl, or ≥300 ng/dl with high LH and FSH were taken as markers of hypogonadism. Data were analyzed using STATA version 15.

**Results:**

A total of 213 participants were enrolled in this study. Between individual’s pairs, the median levels of TT and LH after six and twelve months were found to be significantly higher while estradiol was significantly lower than at baseline (p < 0.001). Between after six and after twelve months, only median estradiol levels showed significant change with levels being lower after twelve months (p < 0.001). The change in FSH was not statistically significant.

Of the 89 participants (41.8%) who had hypogonadism at baseline, 44 (49.4%) normalized TT (≥300 ng/ml) and had higher median testosterone than those who did not normalize. Furthermore, there was a statistically significant association between testosterone change and initial viral load (p = 0.049), WHO clinical stage (p = 0.031) and baseline hypogonadism status (p = 0.014).

**Conclusion:**

This study concludes that TT improved significantly after ART initiation. Particularly, half of the MLWH who presented with low TT at baseline normalized it within the first year of treatment. Therefore, ART reduces prevalence of hypogonadism and baseline TT seems to be predictive of future evolution of the hypogonadism.

## Background

Male hypogonadism is very common among MLWH, reaching a prevalence of up to 89% [1–4]. The most common form of hypogonadism diagnosed in MLWH is secondary hypogonadism characterized by low or inappropriately normal serum LH in presence of serum testosterone below the normal range (i.e., hypogonadotropic hypogonadism) [21,26–29]. With the high prevalence of hypogonadism in MLWH and the early onset of the disease [26,30,31], testosterone deficiency is expected to impact on several aspects of general health and well-being in MLWH including bone health, sexual life and physical performance and may therefore be of concern since most of them are common in MLWH independent of testosterone deficiency [21]. The exact cause of hypogonadism in HIV patients is not known, but decreased gonadotropin release from the pituitary is thought to be one of the mechanisms [32]. There can be other contributory factors for hypogonadism including: aging [33,34], noninfectious comorbidities (obesity, diabetes, hypertension, cancers, malnutrition among others) [31,34–37], anemia [34,35], common acute and chronic illnesses [38], weight loss [5], invasion of glands (testes and pituitary) by HIV, or other pathogens like hepatitis virus [39], cigarette smoking, using drugs, such as opiates, megestrol acetate, methadone [34,40], and steroids [41], as well as progression to acquired immune deficiency syndrome (AIDS) stages [7].

According to findings of studies, hypogonadism has decreased in the antiretroviral therapy (ART) era by about 20% as compared to the pre-ART era. The previous studies done among ART naïve HIV infected males reported prevalence of hypogonadism ranging from 29 to 89.7% [1–4] while those studies among HIV males on ART reporting prevalence ranging from 16 to 34% [5,6]. The effect of ART on hormone levels is not clear [7,8–13] and some studies have reported conflicting results. For example a study by Dube et al [10] and Yoshino et al [14] reported an increase in free testosterone (FT) after initiation of ART while Wunder et al [11] did not find significant changes in FT, LH, or FSH after 2 years of successful ART. Many studies on the prevalence of gonadal dysfunction in HIV, have serious limitations including diagnosis (hypogonadism) on a single, untimed, hormone estimation, inadequate drug history and failure to evaluate the condition clinically. Drug use other than Antiretrovirals (ARVs), both prescribed and non-prescribed like antibiotics, antifungals and chemotherapeutic agents, often used in AIDS patients for controlling several opportunistic infections can also have consequences on endocrine function [15]. There are some comorbidities which also may cause hypogonadism such as diabetes mellitus [16], pulmonary tuberculosis [17,18], Cytomegalovirus [19], *Mycobacterium avium complex* (MAC) infections [20], *Cryptococcus neoformans* infections and infiltrating neoplasms like Kaposi’s sarcoma [19–24]. In addition, the data on changes in gonadal hormones are sparse, especially in recent setting in which an integrase strand transfer inhibitor drugs are used in the antiretroviral therapy regimen [10,11,14,25]. The aim of the present study was to determine changes in gonadal hormones (TT, LH, FSH and estradiol) levels at six and twelve months after initiation of ART.

## Materials and methods

### Study design, setting and subjects

We conducted a longitudinal study on gonadal hormone abnormality among newly diagnosed MLWH starting ART in Mwanza, Tanzania. Newly diagnosed HIV positive (diagnosed as per WHO guidelines 2015) males aged 18 years and above attending at Voluntary Counseling and Testing (VCT) centres at Sekou-Toure Regional Referral Hospital (STRRH), Bugando Medical Centre (BMC), Nyamagana District Hospital (NDH), Magu District Hospital (MDH) and Ilemela District hospital (IDH) were enrolled in the study in a period between January 2020 and August 2022. Patients with previous history of gonadal dysfunction, testicular retention or trauma, taking drugs known to affect hormone levels in the previous six months (i.e., androgens, dehydroepiandrosterone, antiandrogens, anabolic steroids, gonadotropin-releasing hormone (GnRH) agonists and psycholeptic agents [antipsychotics, barbiturates, opiates]), having chronic liver disease, coinfection with hepatitis B virus (HBV) and hepatitis C virus (HCV), chronic kidney injury, tuberculosis and diabetes mellitus that could serve as confounders were excluded from this study.

Sample size for this follow up study was estimated using a formula for longitudinal study with a single mean (Pagano and Gauvreau, 2000) [42].


$$N={\left(\frac{\left(Z\alpha +Z\beta )(\delta \right)}{\left(\mu 1-\mu 0\right)}\right)}^{2}$$

Where:

N = minimum sample size for each group

Zα = percentage point of normal distribution corresponding to the (two-sided) significance level. In this case, significance level will be 5%, so Zα = 1.96

Zβ= Power of the test, which is conventionally 80%; Zβ = 0.84

µ_0_ =hypothesized population mean of hormone

µ_1_ = True mean of hormone in HIV infected (Alternate mean)

The minimum sample size was obtained using the value of mean (SD) testosterone (µ_0_) for non-HIV infected individuals in a study done in Nigeria of 4.74 (1.83). Assuming that the mean testosterone in HIV infected (Alternate mean) is 0.5 units less than this, µ_1_ = 4.24 and have the same SD as for non-HIV infected. Substituting the value in the formula gave the minimum sample size of 105 participants. A convenient sampling technique was used to enroll study participants, where subjects were recruited as they were coming at each VCT until the sample size for the study was attained.

### Data collection and laboratory procedure

All participants were assessed clinically by detailed history taking and general physical examination. Socio-demographic data including age, employment status, marital status, herbal medicine use status (whether they used any herbal medicine within the past six months or not) as well as symptoms of hypogonadism (whether experienced the symptoms in the past six months) were collected using a pre-structured questionnaire. Participant’s body weight was measured with minimal clothing using a standard calibrated weighing scale. Height was measured in an upright standing position using a calibrated stadiometer, and BMI was then calculated by the formula weight in kilogram divided by height in meter squared. WC was measured at the approximate midpoint between the lower margin of the last palpable rib and the top of the iliac crest using flexible plastic tape and were calculated as an average of 3 measurements

Blood samples for viral load were collected into EDTA tubes centrifuged at 4000 revolutions per minute for 20 minutes to obtain plasma. Viral load testing was done using a COBAS TaqMan analyzer (Roche Diagnostics, Germany) as per manufacturer’s instructions.

Blood samples for gonadal hormones analysis were collected from each of the study participants between 8.00 AM and 11.00 AM into plain tubes to obtain serum. The serum samples were stored at -20^o^C for not more than 30 days until analyzed. Serum TT, FSH, LH and estradiol levels were estimated using chemiluminescence immunoassay – analyzer (CLIA) model Maglumi 2000 (Snibe Diagnostic, China) according to the principles of CLIA and protocols given by the kit manufacturers. The CLIA kits were obtained from the Snibe Co., Ltd, Shenzhen, China. The CD4 + count was assessed by flow cytometry (Roche Diagnostics). Measurement of gonadal hormones was done at baseline and repeated at six (6) and twelve (12) months after initiation of ART.

Hypogonadism was defined as a serum TT level of < 300ng/dl or a serum TT level of ≥ 300 ng/dl with high FSH (> 12 mlU/L) or LH (> 12 mlU/L) level [26]. Eugonadism was defined as normal TT and normal FSH and LH levels [26]. Compensated hypogonadism was defined as normal TT but high FSH or LH levels. Primary hypogonadism was defined as low TT levels with high FSH and LH, while secondary hypogonadism was defined as low TT with low or normal FSH or LH [26,43].

### Data analysis

Data were cleaned and checked for completeness and consistency and then corrected. The data were coded and entered into Microsoft excel and then transported to STATA software, version 15 (Texas, USA) for analysis. Data were summarized using frequencies, percentages or median with interquartile range (IQR). Testosterone change was grouped as follows: the change in testosterone with variation of < 50 ng/ml (increase or decrease by < 50 ng/ml) was considered as “No change” and change in testosterone with increase by ≥ 50 ng/m was considered as “Increase” while the change with decrease by ≥ 50 ng/ml was considered “Decrease” [44]. Graphical distribution plots and Shapiro-Wilk test were used to assess the normality of data distribution. Changes of hormones (TT, LH, FSH and estradiol) as continuous variables between pairs over time were assessed using Wilcoxon signed-rank test. Association between variables (age, BMI, WC, marital status, herbal medicine use, CD4 count, viral load, clinical stage and hypogonadism status) and changes in testosterone hormone status at six months were determined using Pearson’s Chi-squared test or Fisher’s exact test where appropriate. We used two-sample proportion test to compare the significance of difference in proportion of hypogonadism and its symptoms between baseline and after six months and twelve months following initiation of ART with the hypothesis that the proportion of hypogonadism will be higher at six months and twelve months after ART initiation than at baseline. In all analyses, the significance levels were set at a p value less than 0.05.

### Ethical considerations

The study received ethical clearance from the Joint Catholic University of Health and Allied Sciences and Bugando Medical Center (CUHAS/BMC) research ethics and review committee with ethical clearance certificate number CREC/407/2019. All study participants provided written informed consent before enrollment into the study and identification numbers were used instead of names. All study patients were prescribed ARV and for those whose hypogonadism persisted after ART their gonadal hormones laboratory reports were shared to the clinicians and the patients were advised to report to the clinicians for advice and further management.

## Results

### Socio-demographic characteristics of 213 study participants

A total of 213 newly diagnosed ART naïve MLWH were enrolled in this study. The median age of the participants was 40 [16–20,22–25,42–46] years (range 18–73 years), and the majority age group was 31–45 years (59.2%, 126/213). The medians BMI and WC of the study participants were 21.0 [19.5–23.4] Kg/M^2^ and 80 [75–83] cm respectively whereby 74.4% of participants had BMI between 18.5 and 24.9 Kg/M^2^ (normal) and 58.7% had WC of less than 81 cm (lower) while 39.4% (84/213) had WC range 81–93 cm. Sixty-two percent of participants (62.4%, 133/213) were married, 72.0% (153/213) were self-employed, and the majority, 65.7% (140/213) reported to have not used herbal medicine in the past six months. Majority of participants were in WHO clinical stage 1 (63.9%, 136/213) and had their viral load at levels that could not be detected by the test used (Target not detected) (67.6%, 100/148) (Table 1).

**Table1:** Socio-demographic and clinical characteristics of 213 study participants.

Variable	Frequency (n)/Median	Percent (%)/IQR
**Median age (Years)**	40	[32.0 – 46.0]
**Age groups (Years)**		
18 - 30	31	14.6
31 - 45	126	59.2
≥ 46	56	26.3
**Median BMI (Kg/m**^**2**^)	21.0	[19.5 – 23.4]
**BMI category (Kg/m**^**2**^)		
<18.5	28	13.3
18.5–24.9	157	74.4
25 - 29.9	24	11.4
≥ 30	2	1.0
**Median Waist Circumference (cm)**	80	[75.0 – 83.0]
**Waist circumference category (cm)**		
<81	125	58.7
81–93	84	39.4
94–115	4	1.9
**Marital Status**		
Married	133	62.4
Single	50	23.5
Divorced/separated	30	14.1
**Herbal Medicine use**		
Yes	73	34.3
No	140	65.7
**Employment status**		
Non self employed	45	21.1
Self Employed	153	71.8
Unemployed	15	7.0
**Median CD4**	309	[158.5 – 425.0]
CD4 category*		
<200	44	29.7
300 - 350	42	28.4
>350	62	41.8
Viral load (Initial)**		
Target not detected (TND)	100	67.6
≤ 1000	41	27.7
>1000	7	4.7
**Clinical stage**		
Stage 1	136	63.9
Stage2	35	16.4
Stage 3	30	14.1
Stage 4	12	5.6

^*^Number of participants N = 146.

^**^Number of participants N = 148.

### Comparison of gonadal hormones levels at baseline and different intervals (six months and twelve months) after ART initiation

The paired medians analysis showed that testosterone levels after six months (483 [280–635] ng/ml) and twelve months (526 [380–685]ng/ml) were significantly higher than at baseline (364 [182–534] ng/ml) and (350 [177–534]) ng/ml respectively (p < 0.001). There were significantly higher and lower medians LH levels after six months and twelve months respectively (p < 0.001). The median estradiol was found to be lower after six months (p = 0.013) and twelve months (p < 0.001) than at baseline. Though not significant, the median FSH was also found to be higher after six months and after twelve months than that at baseline (Table 2).

**Table 2:** Comparison of variables between baseline and after six months, baseline and after twelve months and between after six months and after twelve months following ART initiation (N = 213).

	Comparison at baseline and six months		
**Variables**	**At baseline**	**At six months**	**p-value**
Testosterone (ng/ml)	389 [184–540]	483 [280–635]	<0.001
LH (mIU/L)	5.2 [4.0–7.3]	6.1 [4.4–8.2]	0.013
FSH (mIU/L)	4.2 [2.4–6.5]	4.6 [3.0–6.4]	0.309
Estradiol (pg/ml)	93.4 [92.0–161.5]	92.0 [75.7–101.7]	<0.001
	**Comparison at baseline and twelve months**		
**Variables**	**At baseline**	**At twelve months**	**p-value**
Testosterone (ng/ml)	350 [177–534]	526 [380–685]	<0.001
LH (mIU/L)	5.2 [4.0–7.7]	6.8 [4.6–10.0]	<0.001
FSH (mIU/L)	3.9 [2.4–6.6]	4.3 [2.9–6.2]	0.219
Estradiol (pg/ml)	92.0 [92.0–157.2]	48.6 [25.9–92.0]	<0.001
	**Comparison at six months and twelve months**		
**Variables**	**At six months**	**At twelve months**	**p-value**
Testosterone (ng/ml)	493 [340–648]	526 [380–685]	0.242
LH (mIU/L)	5.9 [4.3–8.3]	6.8 [4.6–10.0]	0.045
FSH (mIU/L)	4.5 [2.8–6.6]	4.3 [2.9–6.2]	0.822
Estradiol (pg/ml)	92.0 [48.4–102.2]	48.6 [25.9–92.0]	<0.001

In comparing hormone levels between six months and twelve months, only the median estradiol level was found to show significant change with levels being lower at twelve months than at six months (p < 0.001). After twelve months, though not significant, the median TT and LH levels were higher while median FSH level was lower than the levels at six months (Table 2).

### Pattern and frequency of change in testosterone levels and hypogonadism following ART initiation

**Fig 1:**
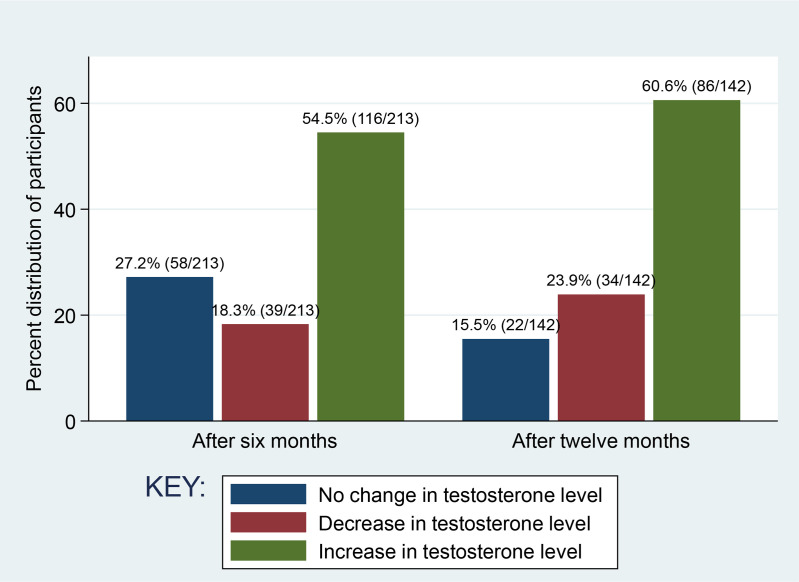
Pattern and frequency of change in testosterone levels among study participants after six months (N = 213) and after twelve months (N = 142) following ART initiation.

After six months, 58 participants (27.2%) had no change in TT (a variation of <50 ng/ml), 41 participants (18.3%) had a decrease (≥ 50 ng/ml) and 116 participants (54.5%) had an increase in TT (≥ 50 ng/mi) (Fig 1). After twelve months, 15.5% (22/142) had no change in TT, 24% (34/142) had a decrease and 60% (86/142) had an increase in TT (Fig 1). There were a total of 89 participants (41.8%) with low testosterone levels at baseline (TT < 300 ng/ml). Of the 89 participants, 81 had low LH (< 12) and low FSH (< 12) (secondary hypogonadism). Fourty four participants normalized TT (≥ 300 ng/ml). Median TT value at baseline of the 44 participants who restored TT was 176 [89.5–228.5] ng/ml, while of those who did not normalize was 138 [92–190] ng/ml. Table 3 presents testosterone change by sociodemographic and clinical characteristics among study participants. There was a statistically significant association between testosterone change of participants and their initial viral load (p = 0.049), WHO clinical stage (p = 0.031) and baseline hypogonadism status (p = 0.014). The majority of participants with testosterone change in increased trend were in clinical stage 1, had hypogonadism at baseline (before ART initiation) and suppressed initial viral load (Table 3).

**Table 3:** Factors associated with change in testosterone levels at six months after ART initiation.

Patient characteristic	Testosterone change			p-value
	Decrease n (%)	No change n (%)	Increase n (%)	
**Age (years)**				0.987^*^
18–30	8 (25.8)	17 (54.8)	6 (19.4)	
31–45	36 (28.6)	68 (54.0)	22 (17.5)	
≥ 46	14 (25.0)	31 (55.4)	11 (19.6)	
**BMI**				0.410^†^
18.5–24.9	31 (19.7)	45 (28.7)	81 (51.6)	
<18.5	5 (17.9)	4 (14.3)	19 (67.8)	
25 - 29.9	2 (8.3)	8 (33.3)	14 (58.3)	
≥ 30 0	0 (0.0)	0 (0.0)	2 (100.0)	
**Waist circumference**				0.373^†^
81–93	11 (13.1)	25 (29.8)	48 (57.1)	
<81	27 (21.6)	33 (26.4)	65 (52.0)	
94–115	1 (25.0)	0 (0.0)	3 (75.0)	
**Marital status**				0.557^*^
Married	26 (19.5)	38 (28.6)	69 (51.9)	
Single	6 (12.0)	12 (24.0)	32 (64.0)	
Divorced	7 (23.3)	8 (26.8)	15 (50.0)	
**Herbal Medicine use**				0.464^*^
No	27 (19.3)	40 (28.6)	73 (52.1)	
Yes	12 (16.4)	18 (24.7)	43 (58.9)	
**CD4 + count**				0.959^*^
>350	11 (17.7)	19 (30.6)	32 (51.6)	
<200	10 (22.7)	14 (31.8)	20 (45.5)	
200–350	9 (21.4)	12 (28.6)	21 (50.0)	
**Viral load**				0.009^†^
Target not detected (TND)	15 (15.0)	22 (21.0)	64 (64.0)	
≤ 1000	5 (12.2)	17 (41.5)	19 (46.3)	
>1000	2 (28.6)	1 (14.3)	4 (57.1)	
**Clinical stage**				0.005^†^
Stage 1	28 (20.6)	34 (25.0)	74 (54.4)	
Stage 2	2 (5.7)	11 (31.4)	22 (62.9)	
Stage 3	3 (10.0)	13 (43.3)	14 (46.7)	
Stage 4	6 (50.0)	0 (8.3)	6 (50.0)	
**Baseline hypogonadism**				0.014^*^
No	27 (24.7)	32 (29.4)	50 (45.9)	
Yes	12 (11.5)	26 (25.0)	66 (63.5)	

^*^ **Chi square.**

^†^ **Fisher’s exact.**

**Fig 2:**
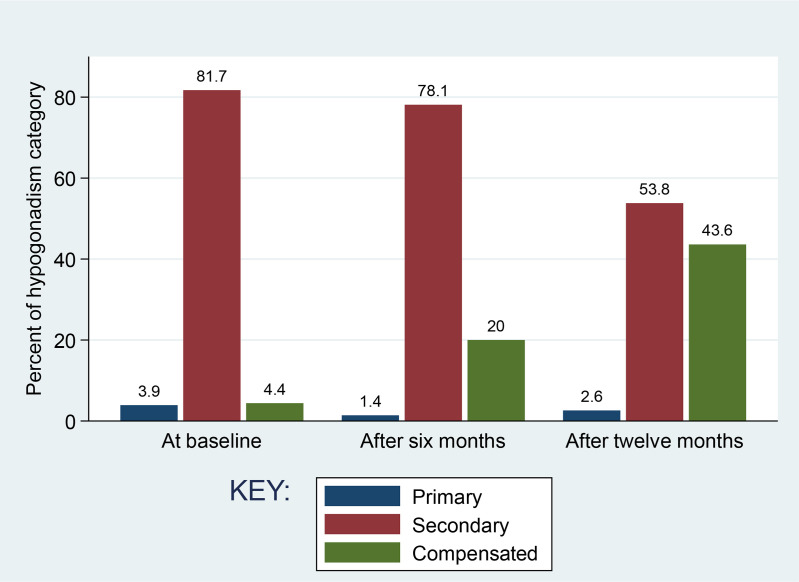
Hypogonadism category (primary, secondary and compensated) distribution among study participants by visits.

The proportion of HIV patients with hypogonadism was significantly lower after six months and after twelve months following ART initiation compared to that at baseline (34.3% [73/213] versus 48% [104/213], p-value 0.001) and (27.5% [39/142] versus 48% [104/213], p-value <0.001) respectively (Two sample proportion test). The proportion of hypogonadism was also lower after twelve months as compared after six months but the difference was not significant (27.5% [39/142] versus 34.3% [73/213], p-value 0.1429) (Two sample proportion test) (Table 4). The secondary type of hypogonadism was the most prominent type at baseline (81.7%, 85/104) and remained to be prominent after six months (78.1%, 57/73) and after twelve months (53.8%, 21/39) following ART initiation (Fig 2)

**Table 4:** Hypogonadism at baseline, after six months and after twelve months among 213 participants.

Hypogonadism	At Baseline	After Six months	After twelve months^*^	p-value six months vs baseline	p-value twelve months vs baseline	p-value twelve months vs six months
	n (%)	n (%)	n (%)			
Yes	104 (48.8)	73 (34.3)	39 (27.5)	0.0012	<0.001	0.1429
No	109 (51.2)	140 (65.7)	103 (72.5)			
**Total**	**213**	**213**	**142**			

^*^ **Total number at twelve months N = 142 (The low number is because some had no hormones results for after twelve months estimation).**

Regarding symptoms of hypogonadism, the proportion of erectile dysfunction (p < 0.001), depression (p < 0.001), weight loss (p < 0.001) and fatigue (p < 0.001), were significantly lower after six months and after twelve months as compared to a baseline (Two-sample proportion test). When comparing between after six months and after twelve months, the proportion of erectile dysfunction (p = 0.049), depression (p = 0.025) and fatigue (p = 0.001) were significantly lower after twelve months as compared to after six months (Two-sample proportion test). The proportion of decreased libido was significantly lower after twelve months (p < 0.001) as compared to after six months but the difference between at baseline and after six months as well as between baseline and after twelve months was not significant (Two sample proportion test) (Table 5).

**Table 5:** Reported symptoms of hypogonadism at baseline, after six months and after twelve months among study participants.

Symptom	At baseline	After six months	After twelve months*	p-value six months vs baseline	p-value twelve months vs baseline	p-value twelve months vs six months
	n (%)	n (%)	n (%)			
**Decrease libido**						
Yes	1 (0.5)	20 (9.4)	0(0.0)	1.000	0.199	<0.001
No	212 (99.5)	193 (90.6)	142 (100)			
**Erectile dysfunction**						
Yes	40 (18.8)	4 (1.0)	0 (0.0)	<0.001	<0.001	0.049
No	173 (82.2)	209 (98.1)	142 (100)			
**Depression**						
Yes	85 (39.9)	9 (4.2)	1 (0.7)	<0.001	<0.001	0.025
No	128 (60.1)	20.4 (95.8)	141 (99.3)			
**Weight gain**						
Yes	6 (2.8)	136 (63.9)	107 (75.3)	<0.001	<0.001	0.011
No	382	77 (36.1)	35 (24.7)			
**Weight loss**						
Yes	147 (69.0)	15 (7.0)	8 (5.6)			
No	66 (31.0)	198 (93.0)	134 (94.4)	<0.001	<0.001	0.299
**Fatigue**						
Yes	167 (78.4)	32 (15.0)	6. (4.2)	<0.001	<0.001	0.001
No	46 (21.6)	181 (85.0)	136 (95.8)			
**Brest enlargement**						
Yes	3 (1.4)	0 (0.0)	0 (0.0)	0.042	0.078	–
No	210 (98.6)	213 (100)	142 (100)			

^*^Number of participants N = 142.

## Discussion

In the present study median TT increased significantly in the following months after initiation of ART with more than half presenting an increase in TT. The serum TT levels increased after ART initiation, especially in the patients who had lower baseline serum TT levels. This finding coincides with the report by Yoshino et al among Japanese treatment-naïve HIV males [14], Dube et al among antiretroviral-naive men [10] and the report by Brigante et al among HIV-positive men with initial finding of low TT [44]. However, the findings are contrary to the report by Wunder et al [11]. The difference in the findings of this study and that of Wunder et al could be contributed by variation in the baseline testosterone levels of study participants.

Previous studies have shown poor health status associated with a worse gonadal function in HIV patients [31,45]. Recent studies have established a link between multi-morbidity, increased body fat (especially abdominal fat), frailty and boosting testosterone decrease [31,45]. The decrease occurs because HIV leads to impaired immunity and hence likely wood of getting opportunistic infections subsequently decrease in testosterone through the described linkage. The boost in testosterone decrease may probably be due to increased aromatization [46] or other mechanisms such as inhibition of gonadotropin secretion or adipokine release [47,48]. The improved TT in the following months after ART initiation in this study might be related to reduced viral load hence improved immunity and halted opportunistic infection.

There was a significant increase in median LH and insignificant decrease in median FSH levels in the following months after ART initiation. These variations might be due to the physiological connection between LH, FSH, estradiol and testosterone. The secretion of testosterone from Leydig cells is regulated by LH and through negative feedback, testosterone reduces the levels of LH and FSH but too low testosterone allows increased secretion of LH and FSH. In a study by Wunder et al the levels of FSH, LH and estradiol did not change significantly after ART initiation [11].

Testosterone in the circulation can undergo peripheral conversion to estrogen by action of Aromatase (estrogenic effect) [49]. Elevated serum LH and FSH concentrations on the testis have a stimulatory effect causing increased conversion of testosterone to estradiol [19]. Also earlier reports have shown occurrence of abnormal androgen metabolism which resulted in increased aromatization of testosterone to estradiol in HIV infected men [50], a fact confirmed by Ezeugwunne *et al*., [51]. The present study showed significantly decreased estradiol level among HIV infected males in the following months after initiation of ART. The decreased estradiol level after ART initiation may be attributable to reduced aromatization as a result of decreased HIV progression by the ART.

More than half of study participants (54.5% and 60.6%) had an increase in TT (More than 50 ng/ml) while about a quarter (27% and 15.5%) had no change in TT (a variation of less than 50 ng/ml) at six months and twelve months respectively after ART initiation. This finding coincides with the report by Brigante et al [44]. The cause of lack of positive change in TT in some participants is unclear but this might be contributed by inadequate adherence to ART and the fact that ART treatment ensures viral suppression and avoids AIDS development [52] but does not fully restore health [52,53].

In the present study subnormal TT levels occurred frequently among ART naïve MLWH (42.2%, 90/213) with most of them having low LH (< 12) and low FSH (< 12) (secondary hypogonadism). This finding corresponds to the previous reports [14,26,44,54–56]. This observation could be due to the invasion of the hypothalamic-pituitary axis by the virus leading to loss of its function. The viral invasion of the hypothalamic pituitary axis may be direct viral infiltration or by systemic or local inflammation resulting in production of pro-inflammatory cytokines such as L-1, IL2, IL-6 and TNF causing hyper or hypofunction [57,58].

Among men with subnormal TT before ART initiation, half of them normalized TT (>300 ng/ml) and had higher median TT value than that of those who did not normalize indicating baseline TT to be predictive of the future evolution of the hypogonadism. This observation coincides with the finding by Yoshino et al [14] and Brigantel et al [44] which showed testosterone increase after HIV treatment especially in patients who had lower serum testosterone levels before ART initiation.

There was a statistically significant association between testosterone change of participants and their initial viral load whereby the majority of participants with testosterone change in increased trend had suppressed initial viral load (< 1000 copies). This finding is in support of the observation in previous studies. A study by Rochira et al [45] found that low serum testosterone is associated with multimorbidity, HIV associated non-AIDS (HANA), and frailty in HIV-infected men (which normally occur when viral load is unsuppressed). Aggarwal et al [59] found lower levels of free testosterone and Dehydroepiandrosterone sulfate (DHEAS) in cases of severe immunosuppression with a statistically significant correlation with CD4 counts. Laudat et al [41] showed serum testosterone, androstenedione and dihydrotestosterone (DHT) levels were decreased significantly in patients with CD4 count < 200/µ (normally occur in unsuppressed viral load). Ferrando et al [60] found serum free testosterone is inversely correlated with viral RNA and confirmed that low serum DHEAS is associated with HIV illness markers, including viral load, and carries negative prognostic value. The high HIV viral RNA viral load will be associated with immunosuppression and subsequent opportunistic infections leading to overall poor health status.

We observed a statistically significant association between testosterone change and WHO clinical stage where the majority of participants with testosterone change in increased trend were in WHO clinical stage 1. HIV infection causes immunosuppression with an associated decrease in CD4 + count leading to comorbid conditions. This observation may also be explained by the link between health status and gonadal hormone secretion. Furthermore, we found that change in testosterone was associated with having hypogonadism at baseline. This is in support of findings by Yoshino et al [14].

Our study findings also showed significant decrease in proportion of hypogonadism after six months (34.3%) and twelve months (27.5%) following ART initiation as compared to baseline (48.8%). This finding is in support of the previous studies which showed reduced range of hypogonadism prevalence among HIV males on ART [5,6] compared to those not on ART [1–4] and in post-ART error [5,6,26,61] as compared to pre-ART error [62,63].

Limitations of the present study include lack of the sex hormone binding globulin (SHBG) measurement, and therefore calculated free serum testosterone which may have resulted in fluctuation of serum testosterone and an underestimation of the prevalence of biochemical hypogonadism since measurement of SHBG has been highly recommended in men with HIV in addition to LH and TT due to the possible rise in serum SHBG in these patients [27,64]. Another limitation is that, testosterone levels were determined using an immune-assay technique, whereas mass-spectroscopy is often considered “gold-standard” but is not commonly used because it is expensive and not widely available. However, the immuno-chemiluminescence assay used in the determination of the gonadal hormone values in this study is internationally certified and widely used in clinical practice to diagnose and guide treatment in patients with gonadal dysfunction. In addition, the study is limited by failure to rule out some disease conditions that may also cause hypogonadism such as Cytomegalovirus (CMV), Mycobacterium avium complex (MAC), Cryptococcus neoformans infections and infiltrating neoplasms like Kaposi’s sarcoma. Further, in the current study, we did not collect some other data which can affect gonadal function such as alcohol use, cigarette smoking and account for them in our study patients.

## Conclusion

This study concludes that TT improved significantly after ART initiation. Particularly, half of the MLWH who presented with low TT at baseline normalized it within the first year of treatment. Baseline TT values seem to be predictive of the future evolution of the hypogonadism, higher TT being associated with subsequent TT normalization. Therefore ART reduces the prevalence of hypogonadism. Further, there was a significant association of testosterone change with suppressed initial viral load, WHO clinical stage 1 and baseline hypogonadism in this study.
